# Height predict incident non-alcoholic fatty liver disease among general adult population in Tianjin, China, independent of body mass index, waist circumference, waist-to-height ratio, and metabolic syndrome

**DOI:** 10.1186/s12889-020-08475-1

**Published:** 2020-03-24

**Authors:** Shubham Kumari, Xuena Wang, Yunyun Liu, Yeqing Gu, Yuhan Huang, Qing Zhang, Li Liu, Ge Meng, Hongmei Wu, Shaomei Sun, Xing Wang, Ming Zhou, Qiyu Jia, Guolin Wang, Kun Song, Kaijun Niu

**Affiliations:** 1 Nutritional Epidemiology Institute and School of Public Health, Tianjin Medical University, 22 Qixiangtai Road, Heping District, Tianjin, 300070 China; 2 Health Management Center, Tianjin Medical University General Hospital, Tianjin, China

**Keywords:** Early life experiences, Height, Insulin-like growth factor-1, Non-alcoholic fatty liver disease

## Abstract

**Background:**

Early-life hormonal and nutritional factors can greatly influence the risk of non-alcoholic fatty liver disease (NAFLD). Adult height is a simple marker for these factors. This study aimed to investigate the association between adult height and NAFLD.

**Methods:**

We performed a prospective cohort study of 35,994 participants aged 25 years or over with measured height at baseline. NAFLD was diagnosed by abdominal ultrasound and self-reported history of alcohol intake. Multivariable Cox proportional hazards regression models were conducted to assess the gender-specific association between height and the risk of NAFLD.

**Results:**

During a follow-up period of 5.5 years, 6245 of 35,994 subjects developed NAFLD. The adjusted hazard ratios (95% confidence interval) of NAFLD for increasing quintiles of height were 1.00 (reference), 0.82 (0.73, 0.92), 0.84 (0.73, 0.97), 0.72 (0.61, 0.85) and 0.63 (0.50, 0.79) (*P* for trend < 0.0001) in males, and 1.00 (reference), 1.00 (reference), 0.80 (0.69, 0.91), 0.72 (0.61, 0.85), 0.60 (0.49, 0.74) and 0.45 (0.35, 0.59) (*P* for trend < 0.0001) in females, respectively.

**Conclusions:**

A higher adult height was associated with lower risk of NAFLD among males and females in Tianjin, China.

## Background

Non-alcoholic fatty liver disease (NAFLD) is defined as the presence of ≥5% of hepatic steatosis, in the absence of secondary causes of hepatic fat accumulation, such as chronic use of medications or significant alcohol intake [1]. It is the most common chronic liver disease all over the world and its prevalence is constantly increasing [2]. About 25% of world population was estimated to have NAFLD [3]. Unhealthy lifestyles and dietary habits in addition to genetic predisposition have pose increased the prevalence of NAFLD in the Asia Pacific region [4].

Early life experiences, including nutrition and hormone, play an important role in influencing later susceptibility to chronic diseases by epigenetic mechanisms [5, 6]. Accumulating evidence suggests that hormonal and nutritional experiences in early life may predispose high incidence of type 2 diabetes mellitus (T2DM) and insulin resistance in later life [7]. Furthermore, there is sufficient evidence to demonstrate that T2DM, insulin resistance and NAFLD share many important metabolic risk factors and common pathogenetic mechanism [8]. Several studies preliminarily suggested that growth hormone (GH) levels and insulin-like growth factor-1 (IGF-1) levels are negatively associated with NAFLD in adults [9, 10], and GH replacement therapy in GH-deficient patients can alleviate NAFLD and improve liver fibrosis [11]. Moreover, suboptimal early life nutrition may increase the susceptibility, age on set, and severity of NAFLD [12].

Adult height is defined as the tallest height after height velocity had decreased to 1 cm or less over 6 months and relatively fixed as compared with child or youth height [13]. Adult height greatly reflects differences in nutrition and hormone levels in early life [14, 15]. It is well recognized that GH and IGF-1 are strongly and positively associated with growth in height, and GH therapy in children with short stature caused by several diseases augments adult height [16]. Since early hormonal and nutritional experiences are major risk factors of final height, it is assumed that adult height may be a potentially useful predictor of incident NAFLD. To date, few studies have focused on the association between adult height and NAFLD.

In the present study, we designed a prospective cohort study to determine whether adult height was associated with the risk of NAFLD among adult population in Tianjin, China.

## Methods

### Study design and participants

Details of the Tianjin Chronic Low-Grade Systemic Inflammation and Health (TCLSIH) Cohort Study have been described elsewhere [17]. Briefly, participants were randomly recruited between January 2010 and December 2016 from the general population in Tianjin, China. The inclusion criteria for the TCLSIH cohort study were men and women who were 18 years and older living in Tianjin, China for at least 5 years. Subjects in the present study were sampled by a random process, using a random number generator. Nearly all occupations are covered in this study, and we also included retired individuals living in residential communities. Therefore, the sample population used here is representative of the general adult population in Tianjin, a typical city in north China. All participants received at least 2 health examinations in our study (including liver ultrasound examination, anthropometric measurements, and blood tests) and completed a structured questionnaire survey [18]. The questionnaire consisted of the following contents: age, gender, smoking and drinking habits, history of diseases (cardiovascular disease, hypertension, hyperlipidemia, and diabetes), and family history of diseases (cardiovascular disease, hypertension, hyperlipidemia, and diabetes). The reliability and validity of the questionnaire have been assessed, with the Spearman’s rank correlation coefficient of 0.67 and 0.58, separately. Written informed consent was obtained from all participants. The ethical protocol of this study was approved by the Medical Ethics Committee of the Tianjin Medical University with the reference number of TMUhMEC 201,430, in accordance with the 1975 Declaration of Helsinki (as revised in 1983).

From 2010 to 2016, a total of 90,536 participants received health examinations. We excluded 170 participants who had missing data on alanine aminotransferase, 4013 participants had excessive alcohol intake (> 140 g/week in males and > 70 g/week in females), and 28,935 participants who had NAFLD at baseline. Moreover, we excluded 767 participants with other liver diseases (including autoimmune liver diseases, chronic hepatitis B or C, cirrhotic or operation on liver), and those with a history of cardiovascular disease (*n* = 5475) or cancer (*n* = 1039), and those aged < 25 years (*n* = 3996). Furthermore, participants were also excluded if they were recruited in 2016 (*n* = 4894) or were lost in following up (*n* = 5253). Furthermore, based on the prevalence of NAFLD in Chinese population and on the principle of 10 outcome events per variable [19], the sample size was calculated. Finally, a total of 35,994 participants were available for analysis (follow-up rate: 87%; followed up for 2–5.5 y; mean duration of follow-up (standard deviation): 2.6 (1.6)).

### Assessment of height

Standing height without shoes was measured to the nearest 0.1 cm using an automatic BMI measuring stadiometer with a precision of 0.1 cm and a range of 0.9–2.50 m (BSM370, Chungcheongnam-do, Korea). In order to investigate how height level is associated with NAFLD, we divided males and female participants into 5 categories (quintiles) according to height, in cm (range), as follows: [1] Level 1 (148.5–167.7), Level 2 (167.8–171.2), Level 3 (171.3–174.3), Level 4 (174.4–178.1), and Level 5 (178.2–204.1) in males [2]; Level 1 (138.0–156.2), Level 2 (156.3–159.5), Level 3 (159.6–162.1), Level 4 (162.2–165.4), and Level 5 (165.5–184.6) in females.

### Diagnosis of NAFLD

Real-time ultrasonography performed by trained and certified technicians was used to diagnose NAFLD. Participants were considered to have NAFLD if [1] they had a self-reported alcohol intake of < 140 g/week and < 70 g/week for males and females, respectively [2]; and at least two of the following abnormal findings of abdominal ultrasound images: diffusely increased liver near field ultrasound echo; increased liver echotexture, compared to the kidneys; vascular blurring and the gradual attenuation of far field ultrasound echo [20]. Inter-observer variations for NAFLD status (yes or no) were evaluated in a subsample of 200 participants from the TCLSIH study. The Kappa coefficient was 0.90, and the total agreement was 96.4%.

### Assessment of other variables

Waist circumference (WC) was measured using a nonelastic plastic anthropometric tape at the level of umbilicus with subjects standing and breathing normally. Waist-to-height ratio was calculated by dividing WC (cm) by the subjects’ height (cm). Participants rest for at least 5 min in a seated position prior to blood pressure measurements. Blood pressure was measured twice from participants’ upper right arms using the TM-2655 device (A&D Company Ltd., Tokyo, Japan), and the blood pressure value was recorded in average. If the first two results are quite different, additional measurements was carried out until stabilization. The mean of the two closest readings (including the last reading) was calculated to determine the reported BP for each participant. Hypertension was finally assessed and diagnosed by physicians according to the criteria of the JNC 8: hypertension was defined as SBP ≥140 mmHg and/or DBP ≥90 mmHg or having history of hypertension or using antihypertensive drugs [21]. Fasting blood samples for the analysis of biochemical values were collected in siliconized vacuum plastic tubes. Fasting blood glucose was measured by the glucose oxidase method, triglycerides were measured by enzymatic methods, low-density lipoprotein cholesterol was measured by the polyvinyl sulfuric acid precipitation method, high-density lipoprotein cholesterol was measured by the chemical precipitation method, and alanine aminotransferase was measured by International Federation of Clinical Chemists (IFCC) method using reagents from Roche Diagnostics on an automatic biochemistry analyzer (Roche Cobas 8000 modular analyzer, Mannheim, Germany). Diabetes was defined as FBG levels ≥7.0 mmol/L or having history of diabetes. Hyperlipidemia was defined as TC ≥5.17 mmol/L or TG ≥1.7 mmol/L or LDL ≥3.37 mmol/L or history of hyperlipidemia. We defined metabolic syndrome (MetS) according to the American Heart Association scientific statements of 2009 [22].

Body weight was measured by an automatic body mass index (BMI) measuring stadiometer (BSM370, Chungcheongnam-do, Korea), accurate to 0.1 kg, with participants wearing only light clothing and no shoes. BMI was calculated as weight (kg) divided by squared height (m^2^). Based on the World Health Organization recommendations for Chinese people, underweight was defined as BMI < 18.5 kg/m^2^, normal weight was defined as 18.5 kg/m^2^ ≤ BMI < 23.0 kg/m^2^, overweight was defined as 23 kg/m^2^ ≤ BMI < 27.5 kg/m^2^, and obesity was defined as BMI ≥ 27.5 kg/m^2^ [23]. Information on family history of cardiovascular disease, hypertension, hyperlipidemia, and diabetes and lifestyle and health-related habits was assessed at baseline using a structured questionnaire. Smoking status was grouped in three: smoker, ex-smoker or nonsmoker and drinking status was classified as everyday, sometime, ex-drinker or nondrinker by self-reporting.

### Statistical analysis

Baseline characteristics of participants were compared using analysis of variance for continuous variables and logistic regression analysis for categorical variables. Continuous variables were shown as geometric mean (95% confidence interval (CI)), and categorical variables were presented as percentage. Cumulative event rates for incident NAFLD were estimated by Kaplan-Meier survival curves, and equalities were compared with the log-rank test.

We tested the interaction between height and the confounding factors, including age, sex, waist circumference, BMI, smoking status, alcohol drinking status, Mets, and family history of disease (cardiovascular disease, hypertension, hyperlipidemia, and diabetes), separately. The interaction between sex and height was statistically significant (*P* <  0.0001), while the *P* values for interaction between height and other confounding factors were > 0.1. Therefore, we analyzed the association between height and NAFLD stratified by sex. We fitted four Cox proportional hazards regression models to evaluate the association between baseline height and incident NAFLD. The initial model was unadjusted model (crude model). Model 2 was adjusted for age and WC. In model 3, we additionally adjusted for smoking status, alcohol drinking status, Mets, family history of cardiovascular disease, hypertension, hyperlipidemia, and diabetes. In model 4, we further adjusted for baseline BMI. In model 5, we further adjusted for baseline waist-to-height ratio. Moreover, we adjusted the history of disease (hypertension, diabetes mellitus, and hyperlipidemia) or the subject’s blood pressure, fasting blood glucose, triglycerides, low-density lipoprotein cholesterol, and high-density lipoprotein cholesterol to replace the metabolic syndrome in the final multiple-adjusted model, separately. All *P* values for linear trends were calculated using the median value for each quintile. All statistical analyses were performed using SAS version 9.4 (SAS Institute, Inc.). Two-tailed *P* <  0.05 was considered as statistically significant.

## Results

**Fig. 1:**
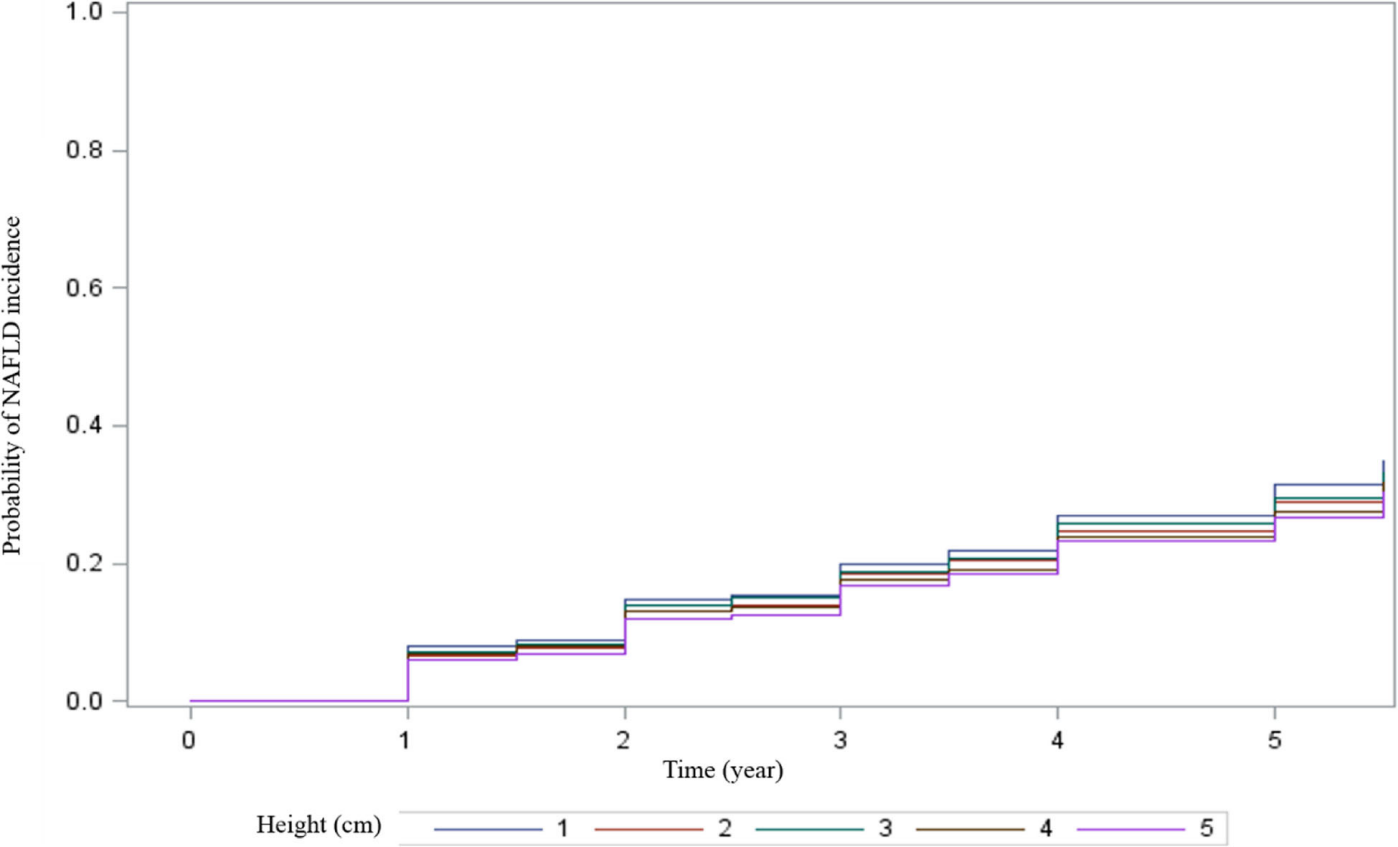
Five-year NAFLD incidence according to baseline height quintiles

In this study, 41.3% of participants were males. Mean age was 42.2 ± 12.9 years for males and 39.2 ± 11.3 years for females. In males, the mean BMI was 24.0 ± 2.9 kg/m^2^, corresponding to 41.8% with BMI category of overweight, and 7.9% with BMI category of obesity. In females, the mean BMI was 22.1 ± 2.9 kg/m^2^, corresponding to 20.3% with BMI category of overweight, and 3.4% with BMI category of obesity. During the 5.5 years of the study period (2010–2016), 6245 of 35,994 individuals (17.4%) developed NAFLD. Incidence of NAFLD was 65.8 per 1000 person-years. The cumulative incidence of NAFLD was significantly lower in subjects in higher quintiles of baseline height compared with those with the lowest quintile (Fig. 1, *P* <  0.0001 by log-rank test). Subgroup analysis showed NAFLD incidence of 110.1, 104.4, 113.2, 108.7 and 110.2 per 1000 person-years in males and 48.4, 43.1, 40.7, 35.9 and 31.7 per 1000 person-years in females, for 1 to 5 height quintiles, respectively.

Characteristics of participants relative to incident NAFLD status for follow-up analysis are presented in Table 1. The mean age (95% CI) in non-NAFLD and NAFLD participants was 38.3 (38.2, 38.4) years and 41.4 (41.1, 41.7) years, respectively. Compared with participants who did not develop NAFLD, participants who developed NAFLD had older age, lower high-density lipoprotein cholesterol, but higher BMI, WC, total cholesterol, low-density lipoprotein cholesterol, triglycerides, fasting blood glucose, systolic blood pressure, diastolic blood pressure, and alanine aminotransferase (all *P* <  0.0001). Participants who developed NAFLD tended to be male, smoker, ex-smoker, everyday drinker, sometimes drinker, while those who did not develop NAFLD tended to be non-smoker and non-drinker (all *P* <  0.0001). In addition, a higher proportion of participants who developed NAFLD had MetS and a family history of cardiovascular disease, hypertension and diabetes (all *P* <  0.0001).

**Table 1:** Baseline characteristics of participants by incident NAFLD status ^a^

	Incident NAFLD		*P* value ^b^
	No	Yes	
No. of subjects	29,749	6245	
Age (y) ^c^	38.3 (38.2, 38.4)	41.4 (41.1, 41.7)	< 0.0001
Sex (males, %)	36.9	62.1	< 0.0001
BMI (kg/m^2^)	22.2 (22.2, 22.3)	24.9 (24.8, 25.0)	< 0.0001
Waist circumference (cm)	75.4 (75.4, 75.5)	83.4 (83.1, 83.6)	< 0.0001
Metabolic syndromes (yes, %)	8.3	26.6	< 0.0001
TC	4.62 (4.62, 4.63)	4.83 (4.81, 4.86)	< 0.0001
LDL	2.65 (2.65, 2.66)	2.90 (2.88, 2.92)	< 0.0001
TG	0.87 (0.87, 0.88)	1.24 (1.22, 1.25)	< 0.0001
HDL	1.48 (1.48, 1.48)	1.28 (1.27, 1.28)	< 0.0001
FBG	4.78 (4.78, 4.78)	4.89 (4.88, 4.91)	< 0.0001
SBP	114.6 (114.6, 114.8)	120.4 (120.1, 120.8)	< 0.0001
DBP	72.1 (72.1, 72.2)	76.1 (75.9, 76.4)	< 0.0001
ALT (U/L)	15.2 (15.2, 15.3)	20.1 (19.8, 20.3)	< 0.0001
Smoking status (%)			–
Smoker	14.3	26.6	< 0.0001
Ex-smoker	2.0	4.3	< 0.0001
Non-smoker	83.7	69.2	< 0.0001
Drinker status (%)			
Everyday	1.6	2.4	< 0.0001
Sometime	37.3	47.4	< 0.0001
Ex-drinker	4.2	4.5	0.27
Non-drinker	56.9	45.7	< 0.0001
Family history of diseases (%)			
CVD	28.1	34.8	< 0.0001
Hypertension	48.0	55.3	< 0.0001
Hyperlipidemia	0.5	0.6	0.055
Diabetes	20.7	26.0	< 0.0001

^a^*NAFLD* non-alcoholic fatty liver disease, *BMI* body mass index, *MS* metabolic syndromes, *TC* total cholesterol, *LDL* low-density lipoprotein cholesterol, *TG* triglycerides; *HDL*, high-density lipoprotein cholesterol, *FBG* fasting blood glucose, *SBP* systolic blood pressure, *DBP* diastolic blood pressure, *ALT* alanine aminotransferase, *CVD* cardiovascular disease

^b^Analysis of variance or logistic regression analysis

^c^Geometric mean (95% confidence interval) (all such values)

Tables 2 and 3 show the crude and adjusted associations between quintiles of height and NAFLD in male and female participants, respectively. In the third multivariate model which didn’t adjust for BMI, the adjusted HRs (95% CI) for NAFLD across height quintiles were 1.00 (reference), 0.86 (0.78, 0.95), 0.90 (0.81, 0.99), 0.77 (0.70, 0.85) and 0.71 (0.64, 0.78) in males and 1.00 (reference), 0.92 (0.82, 1.04), 0.85 (0.75, 0.97), 0.78 (0.68, 0.89) and 0.65 (0.57, 0.74) in females (both *P* for trend < 0.0001). Similarly, in the fourth multivariate model which adjusted BMI, the adjusted HRs (95% CI) for NAFLD across height quintiles were 1.00 (reference), 0.90 (0.81, 0.99), 0.97 (0.87, 1.07), 0.86 (0.78, 0.96) and 0.84 (0.75, 0.94) in males (*P* for trend < 0.01) and 1.00 (reference), 0.97 (0.86, 1.09), 0.98 (0.86, 1.11), 0.93 (0.81, 1.06) and 0.84 (0.73, 0.96) in females (*P* for trend = 0.02). In the final multivariate model which adjusted waist-to-height ratio, the adjusted HRs (95% CI) for NAFLD across height quintiles were 1.00 (reference), 0.82 (0.73, 0.92), 0.84 (0.73, 0.97), 0.72 (0.61, 0.85) and 0.63 (0.50, 0.79) in males (*P* for trend < 0.0001) and 1.00 (reference), 0.80 (0.69, 0.91), 0.72 (0.61, 0.85), 0.60 (0.49, 0.74) and 0.45 (0.35, 0.59) in females (*P* for trend < 0.0001).

**Table 2:** Cohort analysis: adjusted associations of height quintiles with NAFLD ^a^ in males

Cox proportional-hazard regression models	Quintiles of body height (cm, range)					*p* for trend ^b^
	Level 1 (148.5–167.7)	Level 2 (167.8–171.2)	Level 3 (171.3–174.3)	Level 4 (174.4–178.1)	Level 5 (178.2–204.1)	
	(*n* = 3021)	(*n* = 2957)	(*n* = 2852)	(*n* = 3049)	(*n* = 2978)	
Person-years of follow-up	7018	7235	6778	7315	7179	
No. of NAFLD	773	755	767	795	791	
Model 1 ^d^	1.00	0.94 (0.85, 1.04) ^c^	1.02 (0.92, 1.13)	0.97 (0.88, 1.07)	0.99 (0.90, 1.10)	0.75
Model 2 ^e^	1.00	0.87 (0.78, 0.96)	0.90 (0.82, 1.00)	0.77 (0.70, 0.86)	0.71 (0.64, 0.79)	< 0.0001
Model 3 ^f^	1.00	0.86 (0.78, 0.95)	0.90 (0.81, 0.99)	0.77 (0.70, 0.85)	0.71 (0.64, 0.78)	< 0.0001
Model 4 ^g^	1.00	0.90 (0.81, 0.99)	0.97 (0.87, 1.07)	0.86 (0.78, 0.96)	0.84 (0.75, 0.94)	< 0.01
Model 5 ^h^	1.00	0.82 (0.73, 0.92)	0.84 (0.73, 0.97)	0.72 (0.61, 0.85)	0.63 (0.50, 0.79)	< 0.0001

^a^NAFLD, non-alcoholic fatty liver disease

^b^Analysis by Cox proportional hazards model

^c^Adjusted hazard ratios (95% confidence interval) (all such values)

^d^Crude

^e^Adjusted for age and waist circumference

^f^Adjusted for age, waist circumference, smoking status, drinking status, metabolic syndrome, and family history of cardiovascular disease, hypertension, hyperlipidemia, and diabetes

^g^Adjusted for age, body mass index, waist circumference, smoking status, drinking status, metabolic syndrome, and family history of cardiovascular disease, hypertension, hyperlipidemia, and diabetes

^h^Adjusted for age, body mass index, waist-to-height ratio, waist circumference, smoking status, drinking status, metabolic syndrome, and family history of cardiovascular disease, hypertension, hyperlipidemia, and diabetes

**Table 3:** Cohort analysis: adjusted associations of height quintiles with NAFLD ^a^ in females

Cox proportional-hazard regression models	Quintiles of body height (cm, range)					*p* for trend ^b^
	Level 1 (138.0–156.2)	Level 2 (156.3–159.5)	Level 3 (159.6–162.1)	Level 4 (162.2–165.4)	Level 5 (165.5–184.6)	
	(*n* = 4223)	(*n* = 4442)	(*n* = 3947)	(*n* = 4145)	(*n* = 4370)	
Person-years of follow-up	11,318	12,593	11,153	11,576	12,736	
No. of NAFLD	548	543	454	416	404	
Model 1 ^d^	1.00	0.88 (0.78, 0.99) ^c^	0.83 (0.73, 0.94)	0.73 (0.64, 0.83)	0.64 (0.57, 0.73)	< 0.0001
Model 2 ^e^	1.00	0.91 (0.81, 1.02)	0.85 (0.75, 0.96)	0.75 (0.66, 0.85)	0.62 (0.55, 0.71)	< 0.0001
Model 3 ^f^	1.00	0.92 (0.82, 1.04)	0.85 (0.75, 0.97)	0.78 (0.68, 0.89)	0.65 (0.57, 0.74)	< 0.0001
Model 4 ^g^	1.00	0.97 (0.86, 1.09)	0.98 (0.86, 1.11)	0.93 (0.81, 1.06)	0.84 (0.73, 0.96)	0.02
Model 5 ^h^	1.00	0.80 (0.69, 0.91)	0.72 (0.61, 0.85)	0.60 (0.49, 0.74)	0.45 (0.35, 0.59)	< 0.0001

^a^NAFLD, non-alcoholic fatty liver disease

^b^Analysis by Cox proportional hazards model

^c^Adjusted hazard ratios (95% confidence interval) (all such values)

^d^Crude

^e^Adjusted for age and waist circumference

^f^Adjusted for age, waist circumference, smoking status, drinking status, metabolic syndrome, and family history of cardiovascular disease, hypertension, hyperlipidemia, and diabetes

^g^Adjusted for age, body mass index, waist circumference, smoking status, drinking status, metabolic syndrome, and family history of cardiovascular disease, hypertension, hyperlipidemia, and diabetes

^h^Adjusted for age, body mass index, waist-to-height ratio, waist circumference, smoking status, drinking status, metabolic syndrome, and family history of cardiovascular disease, hypertension, hyperlipidemia, and diabetes

When including the history of disease (hypertension, diabetes mellitus, and hyperlipidemia) in the final multiple-adjusted model, the adjusted HRs (95% CI) for NAFLD across height quintiles were 1.00 (reference), 0.85 (0.75, 0.96), 0.87 (0.75, 1.01), 0.76 (0.63, 0.91) and 0.65 (0.51, 0.83) in males (*P* for trend < 0.001) and 1.00 (reference), 0.75 (0.65, 0.87), 0.72 (0.6, 0.87), 0.6 (0.48, 0.75) and 0.44 (0.33, 0.6) in females (*P* for trend < 0.0001). When including the subject’s blood pressure, fasting blood glucose, triglycerides, low-density lipoprotein cholesterol and high-density lipoprotein cholesterol in the final multiple-adjusted model, the adjusted HRs (95% CI) for NAFLD across height quintiles were 1.00 (reference), 0.84 (0.74, 0.96), 0.87 (0.75, 1.02), 0.76 (0.63, 0.92) and 0.64 (0.49, 0.82) in males (*P* for trend < 0.001) and 1.00 (reference), 0.79 (0.67, 0.92), 0.78 (0.64, 0.95), 0.63 (0.49, 0.79) and 0.48 (0.36, 0.66) in females (*P* for trend < 0.0001).

## Discussion

In this large-scale prospective cohort study, we found that higher level of adult height was inversely associated with the risk of NAFLD among males and females in Tianjin, China. The inverse association remained even after controlling for potential confounding factors. To our knowledge, this is the first study to investigate the association between adult height and NAFLD.

We adjusted for multiple potentially confounding factors in our analysis. This study indicated that numerous factors (such as age, BMI, WC, smoking status, metabolic syndrome and family history of some diseases) correlated positively with NAFLD. We used crude model (model 1) first and results showed negative association between height and NAFLD in both males and females. It is well-recognized that NAFLD and height are related to age and WC [24, 25], so we adjusted for these two variables in model 2. Adjustment for age and WC made the associations in males more obvious compared with model 1; however, in females, this adjustment didn’t significantly influence the associations in model 1, leading us to conclude that age and WC are major confounding factors in males but not in females. Since NAFLD was associated with WC, smoking status, drinking status, metabolic syndrome, family history of cardiovascular disease, hypertension, hyperlipidemia and diabetes [26, 27], we additionally adjusted for these variables in model 3. After adjustments for these factors, the associations didn’t change significantly in both males and females, implying these factors may not confound the association between height and NAFLD. In model 4, the present study adjusted BMI and variables in model 3 to confirm the role of BMI in association between height and NAFLD. This adjustment made the association less obvious in both males and females, suggesting that BMI play an important role in association between height and NAFLD. In addition, because waist-to-height ratio has been reported to have a strong association with NAFLD [28], we further adjusted waist-to-height ratio in model 5. The enhanced associations indicated the confounding effect of waist-to-height ratio.

To date, no studies have investigated the association between height and NAFLD. Several studies investigated the association between height and T2DM. Reports about the risk of T2DM and height have produced conflicting results [29, 30]. A meta-analysis showed negative association between height and risk of T2DM in woman only [31], whereas a cohort study of Finnish men showed adult height is associated with decreased risk of T2DM [1]. Since T2DM and NAFLD both result from metabolic dysregulation, these results are, to some extent, consistent with our novel findings that shorter people were associated with higher incidence of NAFLD. Compared to previous cohort studies using Chinese adults with overall incidence of NAFLD ranging from 15.2 to 24.8% [32], the overall incidence of NAFLD in our study is 17.4%, consistent with previous studies.

Although studies of the association between height and NAFLD were sparse, other clinical markers of body composition have been found to be associated with the risk of NAFLD. A prospective study found that the risk of NAFLD increased linearly with increasing BMI [33]. A retrospective cohort study reported that BMI was a useful predictive factor for NAFLD onset [34]. Another study also implied that a greater BMI increase in midlife predicted a greater risk of developing NAFLD [35]. In addition, WC was reported to predict NAFLD with a similar performance with fatty liver index [36]. A population-based study also pointed out that body roundness index and waist-to-height ratio were strongly associated with NAFLD [28]. Results in our current study indicated a substantial inverse association between height and NAFLD risk, regardless of BMI, waist circumference and waist-to-height ratio. Although height is generally non-modifiable, increasing awareness of its potential effects may contribute to the formulation of more accurate risk prediction models and may help subjects to prevent the onset of NAFLD and the development of further complications by changing their other behaviors.

The mechanisms of association between adult height and NAFLD may involve epigenetic changes induced by early life adversity. Interacting with epigenetic mechanisms, hormonal and nutritional conditions in early life influence both attained height and later susceptibility to NAFLD. Although height is shown to be associated with NAFLD, it may be an indicator of risk that likely reflects the hormonal conditions in early life, which are thought to affect health during adulthood [37]. It is well-known that growth hormone (GH) and insulin-like growth factor-I (IGF-I) play essential roles in linear growth [38]. Recently, growing body of evidence has revealed the essential roles of GH and IGF-I in liver metabolism [39]. GH promotes fat metabolism and reduces visceral fat [40], which was closely associated with the progression of NAFLD. IGF-I induces cellular senescence, inactivates hepatic stellate cells, and thus ameliorating fibrosis [41]. Therefore, hormonal conditions in early life may be a potential explanation of the link between greater height and lower NAFLD risk. In addition, data from animal models have indicated that nutritional perturbation of epigenetic regulation is a likely link between prenatal and early postnatal nutrition and health status in later life [42]. A study in mice showed that exposure to prenatal and post-weaning western-style diet predisposed male mouse offspring to the development of NAFLD in adulthood and induced alterations in DNA methylation in key metabolic genes [43]. Biological links, although plausible, remain speculative. Further research is warranted to validate the hypotheses.

The present study has several limitations. Firstly, NAFLD was diagnosed by abdominal ultrasound rather than liver biopsy, which is the gold standard for diagnosis of NAFLD. Ultrasonographic examination is not able to quantify the liver fat or differentiate different stages of NAFLD. Thus, the associations between height and progressive liver disease could not be evaluated in this study. However, abdominal ultrasound is not invasive and widely used in large-scale population-based studies. Moreover, this noninvasive method has a sensitivity of 89% and a specificity of 93% [44]. Second, although we adjusted for a considerable number of potential confounding factors in the present study, residual confounding cannot be excluded. Third, liver mass has been reported to be predicted by the square of height [45]. However, the present study did not measure liver mass, the effect of which on the association between height and the risk of NAFLD cannot be ruled out. Thus, more studies are required to confirm the role of liver mass. Fourth, even though this study population was comprised of general adults in Tianjin, China, the results may be applicable to other similar populations. Fifth, we used the waist circumference measured at the umbilicus in this study. However, waist circumference measured at the iliac crest was reported to be more suitable for use in clinical practice.

## Conclusions

Adult height was negatively associated with the incidence of NAFLD among males and females in Tianjin, China, independent of BMI, WC, waist-to-height ratio, and MetS. The present results indicate that adult height may be a useful predictor for NAFLD to identify high-risk populations and prevent NAFLD at an early age. Such early identification may allow individuals to change their lifestyles to prevent the onset of NAFLD and the development of further complications, which have clinical and public health implications for present and future generations. Future studies are needed to elucidate the mechanism of association between adult height and the risk of NAFLD.

## Data Availability

The datasets generated and analysed during the current study are not publicly available due [public availability would compromise participant privacy] but are available from the corresponding author on reasonable request.

## Abbreviations

BMI: Body mass index
CI: Confidence interval
GH: Growth hormone
HR: Hazard ratio
IFCC: International Federation of Clinical Chemists
IGF-1: Insulin-like growth factor-1
MetS: Metabolic syndrome
NAFLD: Non-alcoholic fatty liver disease
T2DM: Type 2 diabetes mellitus
TCLSIH: Tianjin Chronic Low-Grade Systemic Inflammation and Health
WC: Waist circumference
